# Blood-Brain Barrier Damage in Ischemic Stroke and Its Regulation by Endothelial Mechanotransduction

**DOI:** 10.3389/fphys.2020.605398

**Published:** 2020-12-22

**Authors:** Keqing Nian, Ian C. Harding, Ira M. Herman, Eno E. Ebong

**Affiliations:** ^1^Department of Bioengineering, Northeastern University, Boston, MA, United States; ^2^Department of Development, Molecular, and Chemical Biology, Tufts Sackler School of Graduate Biomedical Sciences, Boston, MA, United States; ^3^Center for Innovations in Wound Healing Research, Tufts University School of Medicine, Boston, MA, United States; ^4^Department of Chemical Engineering, Northeastern University, Boston, MA, United States; ^5^Department of Neuroscience, Albert Einstein College of Medicine, New York, NY, United States

**Keywords:** blood-brain barrier, ischemic stroke, endothelial cells, mechanotransduction, neuroprotection, neurovascular unit, endothelial glycocalyx

## Abstract

Ischemic stroke, a major cause of mortality in the United States, often contributes to disruption of the blood-brain barrier (BBB). The BBB along with its supportive cells, collectively referred to as the “neurovascular unit,” is the brain’s multicellular microvasculature that bi-directionally regulates the transport of blood, ions, oxygen, and cells from the circulation into the brain. It is thus vital for the maintenance of central nervous system homeostasis. BBB disruption, which is associated with the altered expression of tight junction proteins and BBB transporters, is believed to exacerbate brain injury caused by ischemic stroke and limits the therapeutic potential of current clinical therapies, such as recombinant tissue plasminogen activator. Accumulating evidence suggests that endothelial mechanobiology, the conversion of mechanical forces into biochemical signals, helps regulate function of the peripheral vasculature and may similarly maintain BBB integrity. For example, the endothelial glycocalyx (GCX), a glycoprotein-proteoglycan layer extending into the lumen of bloods vessel, is abundantly expressed on endothelial cells of the BBB and has been shown to regulate BBB permeability. In this review, we will focus on our understanding of the mechanisms underlying BBB damage after ischemic stroke, highlighting current and potential future novel pharmacological strategies for BBB protection and recovery. Finally, we will address the current knowledge of endothelial mechanotransduction in BBB maintenance, specifically focusing on a potential role of the endothelial GCX.

## Introduction

Despite significant progression in treatment strategies, stroke remains one of the most common causes of death worldwide and accounts for 55% of all neurological disabilities (Posada-Duque et al., 2014). In the United States alone, approximately 800,000 people suffer from stroke yearly, accounting for $34 billion in medical expenses and lost productivity (Kassner and Merali, 2015; Shi et al., 2016). The majority of observed strokes are classified as ischemic strokes, which occur when a clot within a blood vessel interrupts the cerebral blood flow that supplies vital ions, oxygen, and nutrients to the brain (Obermeier et al., 2013). It is well established that one of the hallmarks of acute ischemic stroke is the disruption of the blood-brain barrier (BBB; Sifat et al., 2017), which leads to the subsequent disruption of ion homeostasis and transporter functions in the brain. The mechanisms of BBB damage in the setting of stroke include modification of tight junction (TJ) proteins, modulation of transporters expression, and inflammatory damage (Abdullahi et al., 2018). Structural injury of TJs combined with BBB transporter dysfunction can collectively lead to increased paracellular solute permeability, ultimately resulting in tissue edema and exacerbating brain injury associated with cognitive impairment (Abdullahi et al., 2018). Therefore, it is necessary to develop therapeutic strategies protecting against BBB dysfunction.

One area of BBB maintenance that has received limited attention is its regulation *via* endothelial mechanotransduction, the conversion of mechanical forces into endothelial cell (EC) biochemical signals (Lam et al., 2012). In the peripheral vasculature, endothelial mechanotransduction has been shown to support proper vessel function *via* the regulation of inflammation, vessel tone, and permeability (Chien, 2008; Jones, 2010; Glen et al., 2012). However, while several studies have demonstrated the presence and function of endothelial mechanotransducers in the BBB (Zaragoza et al., 2012; Conway et al., 2013), more research is necessary to determine the extent and mechanisms by which endothelial mechanotransduction precisely regulates BBB function.

In this review, we first highlight the current understanding of BBB structure. Then, we describe ischemic stroke and its relationship with BBB dysfunction, noting current and potential future therapeutic strategies. Finally, we will put emphasis on studies implicating endothelial mechanotransduction in BBB function and its possibility as a target for novel approaches for BBB protection.

## BBB: Structure and Function

### The Neurovascular Unit

**Figure 1:**
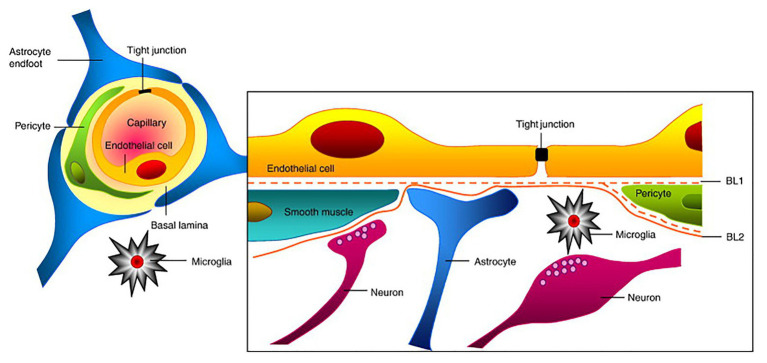
Structure of the neurovascular unit (NVU). Endothelial cells (ECs) form the inner most layer of the NVU and are connected to each other *via* tight junctions (TJs). Surrounding the ECs are pericytes in the case of capillaries or smooth muscle cells in the case of larger cerebrovasculature such as veins and arteries. Additionally, astrocytes and neurons of the NVU extend foot-processes or axons, respectively, that also help regulate NVU function. Finally, microglia, which are the brain’s resident immune cells, also contribute to NVU regulation. Collectively, ECs and these supportive cells help maintain the proper function of the blood-brain barrier (BBB). This figure is reprinted from Abbott et al. (2010)) “Structure and function of the blood-brain barrier,” Neurobiology of Disease, 37 (1): 12–25, with permission from Elsevier.

The blood-brain barrier is an important dynamic and metabolic interface that precisely regulates ion homeostasis in the central nervous system (CNS) and protects delicate neural tissue from potentially toxic substances and pathogens (Jiang et al., 2018). For example, the BBB controls the entry and exit of essential nutrients and waste materials *via* the expression of various channels and transporters to regulate CNS concentrations of ions, neurotransmitters, neuroactive agents, and many other molecules (Jeong et al., 2006). Typically, the BBB primarily refers to the endothelium of the CNS vasculature. However, the development and maintenance of the BBB not only requires ECs and their associated TJs but also requires supporting pericytes, astrocytes, neurons, and extracellular matrix (ECM) that surround the BBB (Table 1). This intricate cellular grouping is referred to as the “neurovascular unit (NVU)” and each cell within the NVU helps promote the proper function of the BBB (Figure 1; Obermeier et al., 2013). Here, we will discuss the function of each cell type in relation to BBB function.

**Table 1:** Components and their functions of the NVU.

Component	Function	Reference
Endothelial cells (ECs)	Main permeability regulators of the neurovascular unit. Possess increased mitochondrial content to increase production of biological energy. Characterized by a lack of fenestrations, minimal pinocytotic activity, enhanced receptor-mediated endocytosis, and increased presence of tight junction proteins, which help with permeability regulation.	Bicker et al., 2014; Knowland et al., 2014
Pericytes	Regulate blood flow and vascular permeability *via*, for example, the induction of occludin and multidrug resistance-associated protein (MRP) expression. Also regulate migration of immune cells. Self-renew and differentiate into neural and vascular lineage cells in the setting of stroke.	Daneman et al., 2010; Dalkara et al., 2011; Nakagomi et al., 2015; Rustenhoven et al., 2017
Astrocytes	Closely interact with ECs *via* astrocyte end feet. Regulate cerebral blood flow and promote BBB function through the release of bioactive substances and regulatory factors, including sonic hedgehog, nitric oxide, and vascular endothelial growth factor. Express the water channel aquaporin 4 (AQP4), assisting with permeability regulation.	Ishida et al., 2006; Iadecola and Nedergaard, 2007; Davis et al., 2010; Alvarez et al., 2013; Liu and Chopp, 2016
Neurons	Communicate with astrocytes to regulate vascular tone and cerebral blood flow.	Koehler et al., 2006
Microglia	Resident immune cell of the brain. Microglia activation contributes to BBB dysfunction.	da Fonseca et al., 2014; Zhou et al., 2006
Extracellular matrix (ECM)	Connects and separates ECs from pericytes and astrocytes to allow proper cellular orientation. Mediates the movement of cells and helps maintain brain homeostasis. The basement membrane of the extracellular matrix connects ECs to astrocytic end-feet to help maintain BBB integrity.	del Zoppo and Milner, 2006; Sandoval and Witt, 2008; Zobel et al., 2016

While ECs of the BBB largely mimic their counterparts in the peripheral vasculature, BBB endothelium also present several phenotypic differences that make them ideal for permeability regulation. For example, BBB ECs have increased mitochondrial content leading to an increased production of biological energy. This energy is required to strictly regulate transport processes through the expression of many receptors and ion channels. Additionally, BBB ECs are characterized by a lack of fenestrations, minimal pinocytotic activity with a small number of endocytotic vesicles, unique receptor-mediated endocytosis, and a heightened presence of TJ proteins. Collectively, these properties help restrict BBB permeability (Bicker et al., 2014; Knowland et al., 2014).

Pericytes of the NVU are located along the basement membrane of BBB ECs, encircling the vessel wall and promoting overall BBB function (Figure 1; Dalkara et al., 2011). For example, pericytes induce occludin and multidrug resistance-associated protein (MRP) expression in ECs and contribute to blood flow regulation *via* their contractile nature. Pericytes also exist in the peripheral vasculature, but the CNS microvasculature has the highest degree of pericyte coverage, potentially contributing to the observed vascular permeability and small vessel stability (Daneman et al., 2010). In addition to their impact on BBB EC function, pericytes exhibit phagocytic activity relevant to the clearance of toxic proteins and can also regulate the influx of immune cells into the nervous system (Rustenhoven et al., 2017). They also display an ability to self-renew and differentiate into neural and vascular lineage cells in the setting of stroke, thereby functioning as pluripotent stem cells (Nakagomi et al., 2015).

Another NVU cell type implicated in promoting BBB function are astrocytes. Astrocytes are the most common glial cell in the brain and have a variety of different morphologies and phenotypes dependent on their location within the brain and association with other cell types. Their close interaction with ECs within the BBB, particularly *via* astrocyte end feet, strengthens the regulation and maturation of the BBB and has been shown to contribute to cerebral blood flow control (Liu and Chopp, 2016). For example, astrocytes release many bioactive substances and regulatory factors that promote BBB function (Alvarez et al., 2013) including sonic hedgehog (Shh), which regulates TJ development and BBB permeability; nitric oxide (NO), which regulates vasodilation (Iadecola and Nedergaard, 2007); and vascular endothelial growth factor (VEGF), which is involved in angiogenesis and vasogenic edema during stroke (Davis et al., 2010). As previously mentioned, astrocytes regulate EC function mainly through their astrocytic end-feet, which extend from their cell body and connect to the basolateral surface of ECs (Figure 1). One astrocyte end-foot protein strongly implicated in BBB function is the water channel aquaporin 4 (AQP4). AQP4, which is involved in the pathogenesis of cerebral edema, facilitates water movement through the plasma membrane of several cell types in the brain, including ECs, and therefore contributes to permeability regulation (Ishida et al., 2006). Additionally, astrocytes play a substantial role in mediating neuroinflammation and thus are significant in neuroinflammatory pathologies including ischemic stroke (Colombo and Farina, 2016).

Neurons have also been reported to regulate BBB function. Although, while direct neuronal contact with the endothelium has been implicated, the incorporation of neurons into the NVU is thought to mainly occur *via* astrocytes. When necessary, neurons will communicate with astrocytes to influence BBB function. For instance, neurons have been suggested to tightly regulate vascular tone and cerebral blood flow *via* astrocytes, which is justifiable given the metabolic demands of nervous tissue; however, neuronal contributions to other BBB structures and function, such as TJ regulation, remain unknown (Koehler et al., 2006).

Although a non-cellular component, the ECM, which is composed of several major proteins including hyaluronan, lecticans, collagen IV, and fibronectin, is an integral component of the BBB and NVU. The ECM functions by connecting and functionally separating brain capillary ECs from pericytes and astrocytes, thereby allowing proper cellular orientation (Zobel et al., 2016). In addition, the ECM mediates the movement of cells within and outside of the NVU and maintains brain homeostasis due to its buffering properties. It is well established that disruption of the ECM and alterations in matrix adhesion receptors, such as integrins, contributes to increased BBB permeability during stroke (del Zoppo and Milner, 2006; Sandoval and Witt, 2008). The basement membrane, a thin layer of ECM that lines the parenchymal side of NVU, connects ECs to astrocytic end-feet and has been implicated in BBB maintenance. Specifically, damage of the basement membrane caused by increased expression of matrix metalloproteinases (MMPs) is believed to be related to alterations in BBB permeability in numerous pathologies (Wang and Shuaib, 2007; Sandoval and Witt, 2008; Lau et al., 2013).

Finally, the NVU also contains microglia, which are the primary immune cells of the CNS (Graeber and Streit, 2010). While the impact of microglia on BBB function in physiological conditions is not well known, activation of microglia *via* neuronal or BBB damage has been shown to contribute to and exacerbate BBB dysfunction (da Fonseca et al., 2014; Thurgur and Pinteaux, 2019). For example, microglial activation resulting in the release of pro-inflammatory cytokines, such as IL-1β and TNF-α, can lead to increased BBB permeability *via* disrupted TJs and increased neutrophil recruitment (Zhou et al., 2006; Chen et al., 2019). Thus, microglia are important to consider when discussing CNS pathologies associated with neuroinflammation.

### Endothelial Cell Junctions

#### Tight Junctions

**Figure 2:**
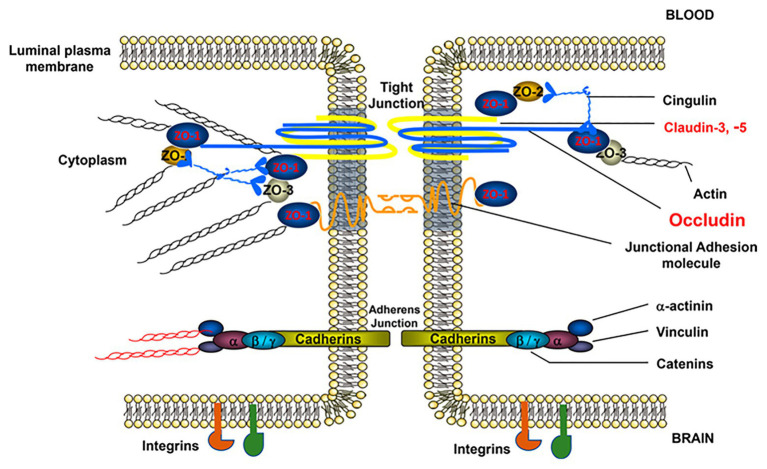
Structure of brain EC-cell junctions. EC-to-EC junctions consist of permeability regulating protein complexes in both tight and adherens junctions (AJs). TJs, which are more strongly associated with permeability regulation, consist of the occludin, junctional adhesion molecule, and claudin families of proteins linked to the cytoskeleton *via* zona occludens (ZO) proteins. AJs, which also play a role in permeability regulation, are composed of vascular endothelial (VE)-cadherin dimers similarly linked to the cytoskeleton *via* an array of linker proteins, such as catenin. In addition to these major junctional components, EC junctions also contain numerous supporting proteins such as cingulin, vinculin, and integrins, among others. This figure is reprinted from a previous publication (Abdullahi et al., 2018), with permission from the previous publisher, the American Physiological Society.

Tight junctions are large, multiprotein junctional cell-cell complexes composed of three major membrane proteins, claudins, occludin, and junction adhesion molecules (JAMs), along with the accessory proteins zonula occludins (ZO; Figure 2). TJs are particularly significant in regulating permeability by inducing EC polarization and restricting the paracellular movement of ions, such as Na^+^ and Cl^−^, as well as macromolecules across the BBB. TJ efficacy in the BBB, specifically compared to the peripheral vasculature, is evidenced by a considerably high value of BBB transendothelial electrical resistance (TEER) and low paracellular permeability (Ronaldson and Davis, 2015).

The tight junction protein family claudins are 20–24-kDa proteins with four transmembrane domains (Figure 2). To date, more than 24 members have been identified (Ballabh et al., 2004). However, in brain ECs, the major isoforms are claudin-1, -3, -5 and -12, all of which play important roles in barrier function (Yang and Rosenberg, 2011). Claudin-5, in particular, significantly limits paracellular diffusion of small substances in the BBB (Greene et al., 2019). Specifically, increased claudin-5 can increase TEER and decrease BBB permeability. To regulate TJ function, the carboxy terminal (intracellular tail) of claudins bind to cytoplasmic proteins including ZO-1, ZO-2, and ZO-3, forming TJ scaffolds between extracellular neighboring proteins and the cell cytoskeleton, thus providing an anchorage to maintain cell-cell contacts (Figure 2; Honda et al., 2006).

A second major transmembrane TJ protein is occludin, a 65-kDa protein involved in TJ stabilization to regulate BBB functional integrity (Figure 2; Nag et al., 2007). Similar to other TJ proteins, occludin is highly expressed in cerebral endothelium but more sparsely distributed in non-neural endothelia (Nag et al., 2007) and is associated with increased electrical resistance in TJ-containing tissues and decreased paracellular diffusion (Nag et al., 2007). As with claudins, ZO proteins also localize occludin to the cellular membrane, creating an intercellular bridge (Figure 2). Interestingly, the function of occludins is suggested to be a function of dynamic phosphorylation, which can affect the assembly/disassembly of TJs (Dorfel and Huber, 2012). Combined with claudins and junctional adhesion molecules, occludins within the BBB strictly regulate BBB permeability to promote CNS homeostasis.

#### Adherens Junctions

Adherens junctions (AJs), located toward the basolateral side of EC TJs, are adhesive cell-cell interactions composed of cadherins along with their associated proteins, which are important for both the localization and stabilization of AJs (Figure 2). The transmembrane cadherin protein and, more specifically, cadherin-mediated signaling plays a key role in numerous EC processes, including endothelial layer integrity and the organization of microvessels during development (Lampugnani and Dejana, 2007). The regulation of these cadherin functions also relies on other AJ components, namely α-actinin and vinculin (Figure 2), which similarly have been implicated in maintaining BBB function (Abu Taha and Schnittler, 2014). Out of the numerous cadherin proteins in humans, VE-cadherin is specifically expressed by ECs and is required for endothelial survival and stabilization (Tietz and Engelhardt, 2015) and is also associated with paracellular permeability (Beard et al., 2011). Interestingly, despite the correlation between VE-cadherin expression and permeability regulation, one particular study suggested that partial loss of VE-cadherin may lead to long-term stroke protection (Gertz et al., 2016).

### Transporters

Endothelial cells of the BBB express many transporters that, in addition to junctional proteins, play an important role in regulating the transport of endogenous and exogenous materials, such as glucose, which cannot permeate cell-cell junctions. Major transporters include nutrient transporters, ion transporters, and the ATP-binding cassette (ABC) transporters, which will be discussed here (Table 2; Jiang et al., 2018).

**Table 2:** EC transporters and their functions.

Component		Function	Diseased condition	Expression	Reference
Nutrient transporters	Glucose transporter 1 (GLUT1) protein	Regulates glucose level in brain	Hypoxia-ischemia elevates GLUT1 expression, which serves a neuroprotective role by reducing focal ischemia	Disperse	Shi et al., 1997; Vemula et al., 2009; Li et al., 2013
	Sodium-glucose transporter (SGLT) protein	Materials transport; cell depolarization; glucose level maintenance	Increases edema formation and exacerbates cerebral ischemic neuronal injury; inhibition of SGLT during stroke improves outcome		Vemula et al., 2009; Yamazaki et al., 2015; Yamazaki et al., 2016; Sifat et al., 2017
Ion transporters	Na^+^-K^+^-Cl^−^ co-transporter	Maintain CNS ion content	Hypoxia stimulates Na^+^-K^+^-Cl^−^ cotransporter expression leading to brain edema formation	Luminal	Foroutan et al., 2005; Jayakumar and Norenberg, 2010
	Na^+^/K^+^-ATPase		Decreased Na^+^/K^+^-ATPase expression causes the accumulation of Na^+^ leading to endothelial swelling and cytotoxic edema	Abluminal	Babsky et al., 2008; Hladky and Barrand, 2016; Jiang et al., 2018
	Ca^2+^-ATPase		Ca^2+^-ATPase fails to maintain ion homeostasis in the setting of stroke due to ATP loss	Luminal	Jiang et al., 2018
ATP-binding cassette (ABC) transporters	P-glycoprotein (P-gp)	Efflux pumps most notably regulating drug transport	Differentially expressed in neurological disorders	Luminal	Abbott et al., 2010; Qosa et al., 2015; Jiang et al., 2018
	Breast cancer resistance protein (Bcrp)				
	Multidrug resistance-associated proteins (Mrps)				

#### Nutrient Transporters

D-glucose is the main energy source for brain metabolism and a continuous supply is therefore required to maintain normal brain function. The main glucose transporter in the BBB is the glucose transporter 1 (GLUT1) protein (Table 2), which is expressed and localized to brain ECs. Many pathophysiological conditions, including chronic hypoxia and ischemia, are capable of altering glucose transporter expression, including GLUT1. However, whether these changes in expression are beneficial or detrimental to BBB and neuronal function varies (Vemula et al., 2009). For example, one study found that hypoxia-ischemia can elevate GLUT1 expression, which in previous studies has been demonstrated to serve a neuroprotective role by reducing focal ischemic lesion size (Shi et al., 1997; Li et al., 2013). Another type of glucose transporter expressed in the BBB is the sodium-glucose transporter (SGLT) protein (Table 2), which contributes to materials transport and cell depolarization and has been shown to help maintain glucose levels during stroke (Yamazaki et al., 2015). However, contrary to the observed beneficial effects of GLUT1, several studies have shown that SGLT is relevant to increased edema formation and can actually exacerbate cerebral ischemic neuronal injury due to the influx of sodium ions (Yamazaki et al., 2016; Sifat et al., 2017). Other studies have corroborated these findings, suggesting that inhibition of SGLT during stroke may improve stroke outcome (Vemula et al., 2009). Thus, regulation of glucose transporters within the BBB can have complex consequences on CNS homeostasis.

#### Ion Transporters

Ion transporters, which are predominantly expressed at BBB ECs’ abluminal surface, have important effects on vectorial transport across the cell membrane and, subsequently, on CNS ion homeostasis and fluid movement. The major ions in the nervous system are Na^+^, K^+^, Cl^−^, and Ca^2+^, all of which are critical to the regulation of neuronal activity (Hladky and Barrand, 2016). The transport of Na^+^, Cl^−^, and other ions combined with the associated water influx is responsible for approximately 30% of the interstitial fluid in a healthy brain. One common ion transporter is the Na^+^-K^+^-Cl^−^ co-transporter (NKCC; Table 2), which is important in maintaining CNS homeostasis (Jayakumar and Norenberg, 2010). It has been reported that hypoxia can stimulate the expression of the Na^+^-K^+^-Cl^−^ cotransporter, leading to ischemia-induced brain edema formation (Foroutan et al., 2005; Jayakumar and Norenberg, 2010). As such, in stroke patients, the entry of extracellular Na^+^ caused by the alteration of these ion transporters, such as decreased Na^+^/K^+^-ATPase expression and function, has a profound impact on endothelial swelling and cytotoxic edema (Babsky et al., 2008; Hladky and Barrand, 2016). Other ion transporters are also implicated in stroke and BBB dysfunction (Hom et al., 2007; O’Donnell, 2014; Jiang et al., 2018). For example, in an ischemic episode, the Na^+^/K^+^-ATPase and Ca^2+^-ATPase ion transporters (Table 2) fail to conserve ionic balance due to ATP depletion caused by the blockage of oxidative phosphorylation (Jiang et al., 2018). Collectively, these studies highlight the importance of ion transporters in regulating BBB function and in pathophysiological events such as stroke.

#### ATP-Binding Cassette Transporters

The ABC transporters (Table 2), which are located at the EC luminal membrane, are a protein superfamily that transports neurotoxins against concentration gradients, thereby requiring biological energy. This protein superfamily contains 48 members and is divided into seven sub-families (Jiang et al., 2018). Major ABC transporters relevant to the BBB include P-glycoprotein (P-gp; Table 2), which transports a variety of structurally diverse compounds; breast cancer resistance protein (Bcrp; Table 2), which is able to recognize variable organic anions; and MRPs (Table 2), which are associated with cellular efflux of anionic drugs as well as their metabolites (Abbott et al., 2010). While ABC transporters protect the CNS by restricting toxin permeability through the BBB, they have also been found to hinder therapeutic strategies targeting the CNS as they similarly limit the transport of drugs through the BBB. It has also been shown that ABC transporter expression varies in neurological disorders such as Alzheimer’s, Parkinson’s, and stroke (Qosa et al., 2015). Thus, while the role of ABC transporters in both proper BBB function and neurological disorders is complex, it is clear that alterations in their expression can have significant consequences on BBB and neural tissue function.

## Ischemic Stroke

### Ischemic Stroke Pathology

Stroke is the second leading cause of death in the world behind cardiovascular diseases and annually contributes to 5.5 million deaths globally (Alishahi et al., 2019). Stroke incidence has also increased more than 100% in developing countries, suggesting that stroke prevalence will likely increase over the coming years (Tsai et al., 2016). At present, there are two major types of stroke: ischemic strokes and hemorrhagic strokes. In this review, we focus on ischemic strokes.

**Figure 3:**
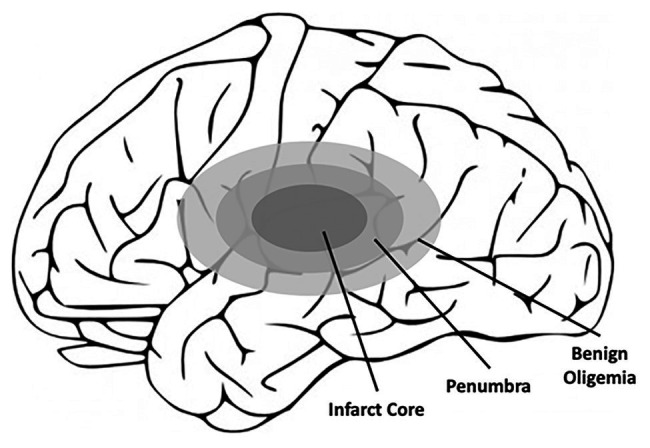
Schematic of the affected areas of the brain after ischemic stroke. Closest to the location of the blood clot, and thus the loss of blood flow, is the infarct core, which contains irreversibly damaged tissue. Outside of the infarct core lies the penumbra, which also contains the benign oligemia, and defines the area of reversible tissue damage. While the benign oligemia specifies tissue that will recover function on its own, tissue within the penumbra but outside of the benign oligemia requires therapeutic intervention for full recovery.

Ischemic strokes amount to approximately 87% of all strokes and are classified as strokes that occur due to vessel obstruction *via* a blood clot (Sevick et al., 2017). These clots can either form directly at the site of insult or result from the rupture of a larger clot at an upstream region of the vasculature. When ischemic strokes occur, blood flow to the brain is interrupted leading to decreased oxygen delivery and reduced nutritional supply (e.g., glucose) to the affected part of the brain (Chauhan and Debette, 2016). The affected areas of the brain resulting from ischemic stroke are referred to as the ischemic, or infarct, core, and penumbra, which collectively form the term “brain ischemia” (Figure 3; Bandera et al., 2006). The ischemic or infarct core is defined as the area of irreversible tissue injury, typically characterized by blood flow with perfusion rates below 10 ml/100 g of tissue per minute (Latchaw et al., 2003; Bandera et al., 2006). Outside of the infarct core is the penumbra, which also contains the benign oligemia (Figure 3), and is defined as functionally impaired tissue that is still viable. Typically, tissue within the penumbra is perfused at rates below 25 ml/100 g tissue per minute (Bandera et al., 2006; Fisher and Bastan, 2012). While tissue within the benign oligemia will recover spontaneously, tissue outside of the benign oligemia but within the penumbra requires clinical intervention. Therefore, therapeutics aimed at preventing CNS damage as result of ischemic stroke target these areas of the brain, whereas the infarct core is beyond treatment due to the rapid development of necrosis (Bandera et al., 2006). The first contributors to the rate of infarct progression are the degree of collateral arterial circulation, duration of insult, and the functional and metabolic cellular state (Bandera et al., 2006). Collectively, these factors determine the likelihood of success of common ischemic stroke therapeutics.

In addition to the initial insults of ischemic stroke, following ischemic stroke metabolic disturbances and energy imbalance can cause secondary injury such as inflammation and gliosis leading to further neuronal and vascular cell death. Inflammation, in particular, is believed to play a critical role in the pathogenesis of ischemic stroke. For example, the initial ischemic insult leads to the activation of microglia (Ma et al., 2017) and astrocytes (Choudhury and Ding, 2016) as well as the recruitment and infiltration of inflammatory cells such as leukocytes (Gronberg et al., 2013). Interestingly, while some studies have indicated beneficial impacts of several of these inflammatory mechanisms on brain recovery, overall the inflammatory response is believed to have a detrimental effect, particularly under prolonged inflammation (Jin et al., 2010). This is supported by the effective use of anti-inflammatory therapeutics to limit neurological deficits in animal models of ischemic stroke (Lakhan et al., 2009), although these therapeutics have not translated into the clinic. These and other therapeutic options for ischemic stroke will be further discussed later in the review.

Although not well studied, endothelial mechanotransduction within the BBB may also play a major role in ischemic stroke pathology and potentially its initial occurrence. In the peripheral vasculature, endothelial exposure to physiological shear stress promotes vessel function through the regulation of vascular permeability (Himburg et al., 2004; Warboys et al., 2010), vasoconstriction/vasodilation (Uematsu et al., 1995; Muller et al., 1997), and inflammatory phenotype (Chen et al., 2003). Recent studies have similarly demonstrated that brain-derived ECs similarly benefit from shear stress exposure *via* observed increases in barrier integrity (Colgan et al., 2007; Siddharthan et al., 2007; Cucullo et al., 2011; Walsh et al., 2011). Thus, stroke-induced hypoperfusion may lead to inadequate mechanotransduction that could further compromise BBB integrity. Additionally, mechanotransducers, such as the endothelial glycocalyx (GCX), have been shown to be degraded in response to ischemia-reperfusion (Rubio-Gayosso et al., 2006), which may exacerbate issues of proper mechanotransduction. Impaired mechanotransduction may even contribute to the occurrence of ischemic stroke, as the development of blood vessel plaques, which can rupture and lead to ischemic stroke, are known to occur in areas of disturbed blood flow characterized by altered mechanotransduction and degraded GCX layers (van den Berg et al., 2009; Cancel et al., 2016; Harding et al., 2018; Mitra et al., 2018). It is therefore vital to understand the impact of endothelial mechanotransduction in both stroke pathology and occurrence, which will be addressed later in the review.

### BBB Dysfunction in Ischemic Stroke

A major hallmark of stroke is its associated BBB disruption, which is initiated due to ischemia but continual deteriorates with sustained hypoperfusion. This deterioration is largely attributed to a lack of nutrients (e.g., oxygen and glucose), but altered mechanotransduction may also play a role. This is evidenced by the fact that even after the restoration of blood flow, albeit below baseline levels, BBB permeability does not revert but instead persists. Furthermore, degradation of the mechanotransductive GCX layer has been shown to be degraded in ischemia/reperfusion (Chappell et al., 2009; van Golen et al., 2012), implicating its potential role in BBB maintenance. The exact time course of increased BBB permeability in ischemic stroke is widely debated. Initial studies in animal models identified a biphasic nature of BBB permeability, in which initial increases in permeability are followed by a reduction in permeability to baseline levels but eventual return to increased permeability (Kuroiwa et al., 1985; Huang et al., 1999). While more recent studies have supported this theory (Pillai et al., 2009), other studies, including in human subjects, suggest that BBB permeability remains elevated post-stroke potentially for several weeks (Strbian et al., 2008; Merali et al., 2017). Regardless, initial breakdown of the BBB is believed to occur at least partially through the overexpression of matrix metalloproteinases (MMPs), which can have numerous detrimental effects including the degradation of the endothelial GCX, a known mechanotransducer (Ramnath et al., 2014; Yang and Rosenberg, 2015; Zhang et al., 2018). GCX degradation has been shown to disrupt junctional protein expression and function (Thi et al., 2004; Mensah et al., 2017). Following this initial breakdown, a sustained increase in permeability likely occurs due to a neuroinflammatory response, which, combined with other consequences such as brain edema, contributes to longer-term permanent loss of neurological function (Abdullahi et al., 2018). To better understand the observed increase in BBB permeability following stroke, we will discuss the mechanisms of this process, focusing on the role of junctional and transporter proteins.

#### Junctional Proteins

The overall increase in BBB permeability in stroke is largely attributed to differences in junctional protein expression and function. For example, stroke is associated with decreased expression of the TJ proteins claudin-5, occludin, and ZO-1, among others (Jiao et al., 2011). This is evidenced by the increased uptake of ^14^C-sucrose, a membrane impermeant marker, in the brain parenchyma after stroke (Hau et al., 2004; McCaffrey et al., 2009; Abdullahi et al., 2018). It has also been suggested that occludin redistribution, in addition to changes in expression, induced by VEGF may occur and is associated with increased paracellular permeability in ECs (Murakami et al., 2009). Caveolin-1, a coat protein of pinocytotic caveolae vesicles implicated in molecular trafficking, mediates the redistribution and localization of junctional proteins, such as ZO-1, claudin-5, and occludin, as previously mentioned (Choi et al., 2016; Abdullahi et al., 2018; Zhang et al., 2018). Additionally, while TJs are more widely known for their role in maintaining BBB permeability, the AJ protein VE-cadherin has also been shown to have decreased expression after stroke, contributing to the overall increase in permeability (Abdullahi et al., 2018). The observed changes in VE-cadherin expression following stroke may be regulated by sphingosine kinase (SphK2), as SphK2-null mice display reduced levels of VE-cadherin and other junctional proteins (Wacker et al., 2012).

**Figure 4:**
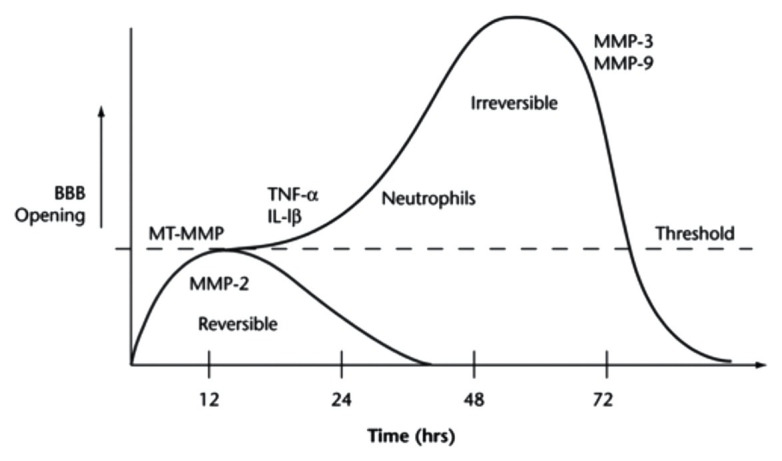
The time course of BBB opening and associated expression of pro-inflammatory mediators of BBB damage. Initial opening of the BBB is likely caused, at least in part, by increases in MMPs, specifically MMP-2. This initial phase occurs over ~24 h and is referred to as “reversible.” Over time, continuous production of pro-inflammatory molecules, such as TNF-alpha, IL-1beta, and MMPs 3 and 9, can lead to prolonged opening (72+ h) of the BBB, potentially contributing to long-term tissue damage. This phase is thus referred to as “irreversible.” Reprinted from Yang and Rosenberg, “Matrix metalloproteinases as therapeutic targets for stroke,” Brain Research, 2015, 1,623:30–38, with permission from Elsevier.

Initial disruption in junctional protein expression is believed to be due to MMPs and, on a lesser basis, reactive oxygen species (ROS). This initial, short-term increase in permeability in some cases reverts to baseline levels and is thus often classified as “reversible” (Figure 4). However, as the molecular response shifts in response to stroke, longer-term BBB opening, referred to as “irreversible,” can persist for several days or longer (Figure 4). What determines the length of increased BBB permeability is not fully known, but molecules such as MMPs and cytokines are implicated in the process. For example, MMP-2 activation is believed to be responsible for initial disruption of TJ proteins (Figure 4). However, in later phases of BBB disruption resulting from stroke, MMP-9 expression is induced and results in more intense and irreversible damage (Figure 4; Yang and Rosenberg, 2015). The role of MMPs in stroke-induced BBB dysfunction was evidenced by Shuai et al., who found that stroke results in decreased ZO-1 expression and translocation of ZO-1 from the cell junctions to the cytoplasm, which occurs due to increased MMP-2/9 and caveolin-1 expression (Zhang et al., 2018). Another study suggested that reduction in brain permeability is highly associated with decreased expression of MMP-2/9 (Zeng et al., 2020). In addition to increased MMP expression and activity, increased concentrations of ROS such as superoxide, which is substantially produced by the NADPH oxidase (Nox) proteins (Revuelta et al., 2019), has similarly been shown to reduce the expression of junctional proteins such as claudin-5 and occludin. For example, Zhanying et al. found that upregulation of TJ protein expression owing to attenuated generation of ROS contributed to improvement of BBB function (Ye et al., 2019). ROS has also been shown to cause caspase-3-mediated damage of TJs and microvascular endothelial hyperpermeability *in vitro* (Alluri et al., 2014). In addition to the short- and long-term impacts of MMPs and ROS on BBB integrity, other inflammatory regulators, such as the cytokines TNF-α (Yang et al., 1999; Pan and Kastin, 2007) and IL-1β (Anthony et al., 1997; Ferrari et al., 2004), as well as neutrophils (Rosell et al., 2008; Joice et al., 2009; Jickling et al., 2015) have been implicated in BBB maintenance following stroke. Due to the proven detrimental effects of these factors, therapeutics targeting these molecules have drawn significant interest to maintain BBB integrity.

#### Endogenous BBB Transporters

Changes in the expression and function of endogenous BBB transporters also impact stroke-induced BBB permeability and the resulting pathophysiological processes such as edema. Major BBB transporters associated with stroke include the glucose transporter proteins and ion transporters. For instance, the GLUT1 and GLUT3 glucose transporters are significantly elevated in the short and long term time periods following severe hypoxic-ischemic insults (Vannucci et al., 1998). Increased expression of the SGLT glucose transporter has also been observed and inhibition of this and the GLUT1 transporter can reduce edema formation despite the beneficial nutrient transport roles of these proteins (Vemula et al., 2009). Also implicated in permeability regulation in stroke and subsequent edema formation are ion transporters, particularly the Na^+^ transporters. For instance, increased expression and phosphorylation of the Na^+^-K^+^-Cl^−^ transporter is associated with severe ischemic conditions (Foroutan et al., 2005). Furthermore, the inhibition of various ion transporters, including Na^+^-K^+^-Cl^−^ (Huang et al., 2019), NHE1 (Park et al., 2010), and KCa3.1 (Chen et al., 2015), have been shown to reduce edema or infarct size, highlighting their importance in stroke. In addition to alternations in specific transporter proteins, elevation of pinocytosis within the endothelium occurs post ischemia-reperfusion (Shinozuka et al., 2013), further contributing to increased permeability (Xia et al., 2012). A study by Knowland et al. (2014) even suggested that increased transcellular transport *via* caveolae and other pinocytotic vesicles occurs before TJ disruption.

#### Other Mechanisms

In addition to the common permeability pathways controlled by junctional proteins and EC transporters, other molecules, such as integrins and EC adhesion molecules, also play a role in regulating BBB permeability after stroke, albeit to a lesser extent. Integrins, transmembrane proteins that interact with the ECM to stabilize ECs to their environment, have been shown to contribute to BBB permeability regulation. For example, Osada et al. (2011) demonstrated that antibody neutralization of b_1_-integrin leads to a significant decrease in BBB barrier integrity, specifically through a reduction in transepithelial electrical resistance and increased permeability. Studies suggest that integrins are able to regulate BBB integrity through both the downstream regulation of TJ proteins, specifically claudin-5 (Osada et al., 2011), and by promoting EC-astrocyte interactions, which are crucial for proper BBB function (Almutairi et al., 2016).

Endothelial cell adhesion molecules, such as intercellular adhesion molecule 1 (ICAM-1), also play a role in regulating transport in the BBB in particular through their role in leukocyte transendothelial migration. After focal brain ischemia, EC adhesion molecules, mainly ICAM-1, are upregulated potentially *via* the local and systemic release of pro-inflammatory factors (Lindsberg et al., 1996). Combined with increased leukocyte production in response to stroke (Ross et al., 2007), this promotes increased transendothelial migration of leukocytes, such as neutrophils. While short term increases in neutrophils and other white blood cells may help resolve vascular and CNS damage, prolonged neutrophil accumulation, and activity actually worsens long term outcomes (Buck et al., 2008; Kumar et al., 2013).

## Therapeutic Strategies for Ischemic Stroke

Current therapeutic strategies for ischemic stroke fall into four categories: clinical care, neuroprotection, neurorestoration strategies, and rehabilitation therapy (Patel and McMullen, 2017). To date, the only major treatment strategies approved by the United States Food and Drug Administration (FDA) and used in the clinic are thrombolysis *via* tissue plasminogen activator (r-TPA) and endovascular treatment. However, while these treatments have been successful in improving clinical outcome, they are not able to resolve prolonged neuronal dysfunction and degeneration of still viable tissue. Therefore, many studies and experimental trials have been focused on developing neuroprotective therapies to retain function of viable tissue after an ischemic episode. Yet, while many animal studies have demonstrated promising results, they have met limited success in clinical trials. In this review, we will first address stroke management in the clinic and then elaborate on both previous and promising neuroprotective treatment options currently being assessed (Table 3). Furthermore, we will briefly discuss neurorestorative therapies.

**Table 3:** Current and promising therapeutics for treatment of ischemic stroke.

Therapy	Functionality	Reference
*Alteplase*	Tissue plasminogen activator used for thrombolysis. Only FDA approved pharmacological treatment. Only utilized within 3–4.5 h of stroke.	Bansal et al., 2013; Sifat et al., 2017; Clarke and Ayala, 2019
*Mechanical thrombectomy*	An FDA approved treatment that utilizes a catheter to navigate blood vessels to the site of the clot for mechanical disruption. Has shown efficacy, but is a difficult procedure and only 10% of patients fit the criteria for treatment.	Mokin et al., 2014; Lapergue et al., 2020
*Cerebrolysin*	A mixture of brain-derived neuropeptides that exhibits anti-excitatory, anti-inflammatory, and anti-apoptotic activity. Approved in over 40 countries outside of the United States.	Goenka et al., 2019
*Magnesium sulfate*	An anti-excitatory treatment designed for neuroprotection. Has demonstrated a mixture of success and failure in clinical trials.	Lampl et al., 2001; Muir et al., 2004; Saver et al., 2015
*Minocycline*	An antibiotic designed for neuroprotection that displays anti-inflammatory and anti-apoptotic properties. It has demonstrated success in animal models but has been met with mediocre success in clinical trials.	Fagan et al., 2010; Karsy et al., 2017
*Edaravone*	An anti-oxidant therapy approved for the treatment of ischemic stroke in Japan, but not the US. Has demonstrated some success in clinical trials.	Kimura et al., 2012; Kikuchi et al., 2013
*DI-3-n-butylphthalide (NBP)*	A synthetic compound with intrinsic anti-inflammatory, anti-oxidative, and anti-apoptotic properties approved for the treatment of stroke in China and currently in clinical trials in the United States.	Li et al., 2019; Wang et al., 2019
*Liraglutide/exenatide*	Glucagon-like peptide-1 agonists that reduce infarct size and improve neurological function in animals. Ongoing clinical trials.	Sato et al., 2013; Zhu et al., 2016
*Neurotophin-3*	A neurorestorative therapy that has demonstrated neurogenesis in animal models but no success in humans.	Vilar and Mira, 2016; Duricki et al., 2019

### Clinical Care

Two major thrombolytic therapeutic strategies, either *via* the use of pharmacological agents or *via* mechanical thrombectomy (Jovin et al., 2015; Saver et al., 2016), are currently used for recanalization and reperfusion for stroke patients (Table 3). The treatment of choice for each patient depends on the time to treatment and the etiology of the injury. At present, the most common pharmacological treatment is thrombolysis *via* r-tPA, known as alteplase (Table 3), which is approved by the FDA (Sifat et al., 2017). When administered intravenously, alteplase provides local fibrinolytic effects by first binding to fibrin clots and then hydrolyzing peptide bonds in surrounding plasminogen, thus converting it to plasmin and subsequently disintegrating the clot (Bansal et al., 2013; Clarke and Ayala, 2019). While studies have demonstrated that alteplase does reduce tissue infarct volume, it does not alter other associated pathophysiologies such as ischemic brain edema (Broocks et al., 2020). While r-tPA has been demonstrated as an efficient and cost-effective thrombolytic agent, its short therapeutic time window of up to 4.5 h, association with severe deleterious events, especially hemorrhagic transformation and brain injury, and candidates’ eligibility for treatment (e.g., delayed identification in the emergency department; Sung et al., 2013) greatly limit the safety and application of r-tPA (Mendez et al., 2018). Regarding deleterious events associated with tPA, it has been proposed that tPA exacerbates neuron death in the hippocampus due to inter-neuronal laminin degradation and pro-survival cell-matrix signaling disruption (Lo et al., 2004). Additionally, evidence suggests that a compromised BBB may limit the efficacy of r-tPA because it may exacerbate hemorrhagic transformation and promote brain edema and neuroinflammation. Due to these limitations, alternatives to alteplase, such as tenecteplase, have been investigated but have not shown any significant improvements in clinical trials (Logallo et al., 2017). Still, it is clear that areas for improvement exist.

Aside from r-TPA, other minor pharmacological treatment options are available, most notably aspirin. Aspirin treatment, which is only used for secondary prophylaxis, must be delivered within 24–48 h after stroke onset but has been reported to lower the potential risk of recurrent stroke and vascular events than non-users (Kong et al., 2019). However, it is suggested to be useless for the treatment of an ongoing acute ischemic stroke and, thus, alternative pharmacological therapeutic options remain the primary mode of treatment (Powers et al., 2018).

In addition to these pharmacological treatment options, endovascular mechanical thrombectomy treatment (Table 3), which utilizes micronavigation of catheters into the cerebral vasculature to mechanically disrupt clots (Akins et al., 2014), provides a more direct way to the occlusive lesion and has a longer therapeutic time window-up to 8 h (Jovin et al., 2015). Furthermore, it is reported that endovascular treatment combined with intravenous pharmacological thrombolysis may provide increased efficacy to many patients with ischemic stroke (Lowhagen Henden et al., 2017). Endovascular mechanical thrombectomy has also shown great benefits in large vessel occlusion *via* a combination of contact aspiration and stent retrievers (Mokin et al., 2014; Lapergue et al., 2020). However, despite the potential benefits, endovascular mechanical thrombectomy treatment is limited by high costs, its need for trained personnel, and its inability to restore tissue function within the penumbra.

### Neuroprotection

Neuroprotective treatments are defined as therapeutics administered during the acute ischemic phase with the overall goal of protecting from further neuronal tissue injury. These therapeutics typically target the penumbra as this tissue is still viable but may require clinical intervention for complete functional recovery. Neuroprotective treatments as defined here are in contrast with neurorestoration techniques, which aim to restore tissue functionality through the stimulation of neurogenesis and neuroplasticity. There are two types of neuroprotection: pharmacologic neuroprotection and non-pharmacologic neuroprotection. Non-pharmacologic neuroprotective methods, such as transcranial laser therapy (Patel and McMullen, 2017), have shown some promise but will not be discussed in this review. For pharmacologic neuroprotection, our understanding of the mechanisms of tissue damage following ischemic stroke has advanced, providing scientists with viable targets for neuroprotective therapeutics. Many developed and tested therapeutics have targeted the modulation of inflammation, oxidative stress, excitotoxicity, or apoptosis, all of which have been implicated in post-stroke tissue damage (Goenka et al., 2019; Wang et al., 2019). However, despite the relatively high levels of therapeutic benefits identified by these treatments in animal models, limited success has been observed in the clinic despite the hundreds of clinical trials run. The low degree of therapeutic translation from animal models to human subjects is likely due to a combination of reasons relating to differences between animal models and human subjects (e.g., age of treatment and presence of comorbidities), the employed experimental methods, the quality of experimental or clinical trial design, the outcome measures utilized, and others. Thus, future research efforts attempting to identify neuroprotective therapeutics not only need to identify the novel therapeutics themselves but also enforce proper pre-clinical and clinical methods to properly evaluate the efficacies of these treatment options. Here, we will discuss several drugs that may prove to be promising therapeutics or may provide insight into the development of other future therapeutics.

While the only FDA-approved pharmacological therapeutic for ischemic stroke is tPA, a variety of other neuroprotective molecules have been approved for clinical use for other indications or in other countries. Still, the therapeutic efficacy of these treatments is controversial. For example, cerebrolysin (Table 3) is a mixture of brain-derived neuropeptide that exhibits anti-excitotoxicity, antioxidant, and anti-apoptotic activity that is approved for the treatment of stroke in more than 40 countries (Goenka et al., 2019). Several animal studies have demonstrated that cerebrolysin can improve outcome from stroke, for example, by reducing infarct size (Ren et al., 2007; Zhang et al., 2010). However, clinical trials for cerebrolysin have returned mixed results. Specifically, while the drug has demonstrated safety in Phase 1 and Phase 2 clinical trials, several later stage trials failed to demonstrate any improvement in neurological outcome. Although, a combination of cerebrolysin and physical rehabilitation therapy was shown to improve motor recovery in individuals with severe motor impairment.

Magnesium sulfate (Table 3), which modulates excitotoxicity, has also demonstrated success in animal studies but has been met with both success and failure in a clinical setting. An initial pilot clinical trial from 2001 found a significant correlation between magnesium sulfate and improved neurological outcome (Lampl et al., 2001). However, another larger future study identified no relationship between the drug and neurological outcomes (Muir et al., 2004). Because the administration time in this study was in some cases significantly delayed, an additional clinical trial utilizing magnesium sulfate treatment in the field within 2 h of stroke onset was therefore performed and demonstrated a significant improvement in neurological outcomes (Saver et al., 2015). Other promising drugs, such as minocycline (Table 3), an antibiotic drug approved in the United States for numerous non-stroke indications, and edaravone (Table 3), an antioxidant therapy, have similarly demonstrated promise in animal trials but mixed results in clinical trials (Fagan et al., 2010; Sharma et al., 2011; Kimura et al., 2012; Kikuchi et al., 2013; Karsy et al., 2017). These studies, among many others, demonstrate both the difficulty of identifying promising stroke therapeutics and the importance of proper pre-clinical and clinical study design. Currently, new therapeutics are being tested in clinical trials around the world that will hopefully prove to be more successful than previous waves.

One such molecule is Dl-3-n-butylphthalide (NBP; Table 3), a synthetic compound that possesses anti-inflammatory, anti-oxidative, and anti-apoptotic properties and has been approved for treatment of ischemic strokes in China (Wang et al., 2019). Animal models evaluating NBP as a stroke therapeutic have found positive results. For example, Wang et al. (2019) identified increased neurological functional recovery after NBP treatment for ischemic stroke in mice, which was associated with increased white matter integrity and the upregulation of the TJ protein occludin. Similarly, Jiamin et al. demonstrated that NBP greatly reduces BBB permeability *via* the upregulation of claudin-5 and ZO-1 and downregulation of caveolin-1 in ischemic stroke mice (Li et al., 2019). These and other results have supported the use of NBP as a therapeutic, which is currently being evaluated in the clinic in a Phase 2 trial. Another promising therapeutic option, liraglutide (Table 3), a glucagon-like peptide-1 receptor agonist, is supported by a significant body of work which has identified both its beneficial effects on neurological function in animal models of stroke and its mechanism. For instance, numerous studies have demonstrated improved behavioral scores/neurological deficits and reduced infarct size in animal models of stroke treated with liraglutide (Sato et al., 2013; Zhu et al., 2016). These improved outcomes are believed to be due to reductions in oxidative stress, improved mitochondrial function, and reduced cell apoptosis *via* neuronal sirtuin 1 and activation of the PI3K/AKT and MAPK pathways (Sato et al., 2013; Zhu et al., 2016; He et al., 2020b). A phase 3 clinical trial evaluating the efficacy of liraglutide for treatment of ischemic stroke is currently underway. Another glucagon-like peptide-1 receptor agonist, exenatide (Table 3), has also met success in animal models and has an ongoing phase 3 clinical trial. Other recent therapeutic options, such as alpha lipoic acid, verapamil, soyateltide, and JPI-289, have also recently demonstrated success in animal models and currently have ongoing clinical trials. However, whether these new therapeutics will have higher success than previous neuroprotective therapies is still yet to be seen.

### Neurorestoration

While neuroprotection focuses on preventing further damage to tissue, neurorestoration aims to restore function to damaged neurons *via* the use of neurotrophins, a group of proteins associated with the maintenance and survival of the CNS. The most abundant neurotrophin in the adult brain is brain-derived neurotrophic factor (BDNF), which participates in proliferation and neuronal differentiation, among other functions (Liu et al., 2020). Another common and well-studied neurotrophin, neurotrophin-3 (NT-3; Table 3), is specifically involved in cell proliferation as well as the processes of memory and learning (Vilar and Mira, 2016). As a stroke therapeutic, NT-3 has demonstrated excellent translational potential. For example, studies have shown that peripheral infusion of NT-3 can increase sensorimotor function after stroke. Additionally, phase 1 and phase 2 clinical trials have already been performed for NT-3, demonstrating its safety as a therapeutic (Duricki et al., 2019). Combined with NT-3’s ability to be transported to the CNS through the BBB, these findings make it a promising future therapeutic. Metformin, another potential neurorestorative therapy, has also been shown to induce recovery of memory and learning by increasing the expression of the neutrophin BDNF (Ghadernezhad et al., 2016). Interestingly, metformin has also been shown to regenerate the GCX (Eskens et al., 2013; Targosz-Korecka et al., 2020). However, whether or not metformin-induced GCX regeneration plays a role in neurorestoration following stroke has not been studied. Other therapies, such as copolymer-1 (Cop-1), can similarly increase NT-3 and other neurotrophins to promote neurogenesis (Cruz et al., 2015). However, while some of these treatments have demonstrated efficacy *in vitro* or in animal models, further research is necessary to support their testing in clinical trials.

## Endothelial Mechanobiology in the Peripheral Vasculature, BBB Maintenance, and Ischemic Stroke

One regulatory mechanism of vascular function that has been well studied in the peripheral vasculature but not in the context of the brain vasculature (i.e., the BBB), neither in physiological nor pathological conditions, is endothelial mechanobiology. Endothelial mechanobiology is the process by which ECs sense mechanical forces and convert these forces into biochemical signals (Davies, 1995; Ando and Yamamoto, 2009). These forces include shear stress created by blood flow and tension created by vessel contraction/dilation and changes in the ECM (He et al., 2020a). In the peripheral vasculature, endothelial mechanobiology promotes proper function of the vasculature by regulating permeability, vascular tone, inflammatory state, and other important vascular functions implicated in vascular disease (Chien, 2008; Jones, 2010; Glen et al., 2012). For example, in the peripheral vasculature, both *in vitro* and *in vivo* studies have demonstrated that exposure to physiological levels of fluid shear stresses strengthens vascular barrier integrity (Himburg et al., 2004; Warboys et al., 2010). *Via* a novel *in vivo*/*ex vivo* permeability technique, Himburg et al. (2004) specifically found that increasing levels of physiological time-averaged shear stress leads to decreased endothelial permeability. Shear stress exposure has also been shown to increase nitric oxide production (Uematsu et al., 1995) and reduce the production of reactive oxygen species (Chen et al., 2003) when compared to samples maintained in static, no-flow conditions. In contrast, impaired mechanotransduction in the peripheral vasculature may promote the development of cardiovascular diseases such as atherosclerosis. In the case of atherosclerosis, a disease characterized by plaque formation in vessel walls, plaque development preferentially occurs in areas of the vasculature that experience abnormal flow patterns. Blood flow in these regions is typically characterized by multi-directional flow and low time-averaged shear stresses, leading to impaired mechanotransduction (Zarins et al., 1983; Ku et al., 1985).

Endothelial mechanotransduction has also been proven beneficial in the BBB. Specifically, several studies have demonstrated that exposure of brain EC monocultures or co-cultures to shear stress leads to increased TEER, reduced permeability, and increased expression of junctional proteins (Colgan et al., 2007; Siddharthan et al., 2007; Cucullo et al., 2011; Walsh et al., 2011). For example, Cucullo et al. (2011) found that shear stress application of 6.2 dynes/cm^2^ to a co-culture of human brain microvascular ECs and astrocytes leads to increased expression of TJ proteins, such as occludin and claudin-5, while simultaneously upregulating protective transporter proteins such as the ABC transporter family. This led to reduced permeability of numerous molecules including mannitol and d-glucose. Although, while this and other studies have correlated shear stress exposure with increased barrier integrity, many of these studies utilize sub-physiological shear stress magnitudes or do not include important supportive cells such as pericytes and astrocytes. Furthermore, while the impacts of shear stress exposure have been demonstrated, the mechanostransducers responsible for the observed impacts are largely unknown. In this section, we will address a prominent endothelial mechanotransducing structure, the GCX, and elaborate upon the potential of this and other mechanotransducers as future therapeutic targets for the treatment of stroke and other diseases associated with BBB dysfunction.

### Endothelial Glycocalyx Structure

**Figure 5:**
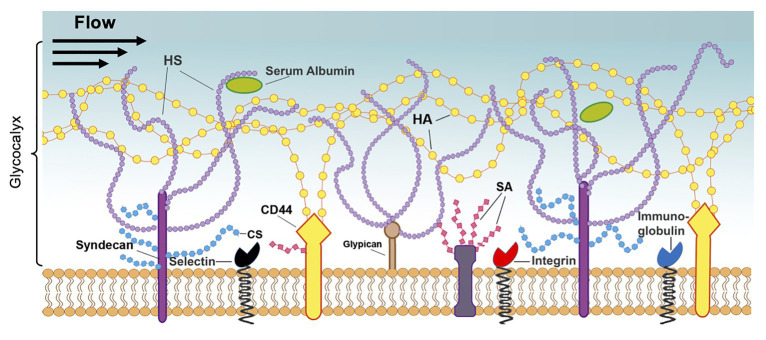
Structure of the endothelial glycocalyx. The glycocalyx, a mechanotransducer implicated in BBB regulation, extends from the membrane of ECs into the vessel lumen and is largely composed of proteoglycans and glycoproteins. The proteoglycans consist of the core proteins, syndecans, and glypicans, along with their attached glycosaminoglycans: heparan sulfate (HS), chondroitin sulfate (CS), and hyaluronic acid (HA). These chains are also modified by sialic acid (SA), which provides a net negative charge to the structure. Additionally, the glycocalyx contains glycoproteins, such as integrins and immunoglobulins, and soluble proteins, such as albumin. Reprinted from Harding et al., 2019 “Endothelial barrier reinforcement relies on flow-regulated glycocalyx, a potential therapeutic target,” Biorheology, 56 (2–3): 131–149, with permission from IOS Press. The publication is available at IOS Press through http://dx.doi.org/10.3233/BIR-180205.

The endothelial GCX is a transmembrane, proteoglycan-glycoprotein layer extending from the luminal surface of ECs with a reported thickness ranging from 20 nm to 11 mm *in vitro* and *in vivo*, depending on the size and location of the vessel, as well as the method of GCX preservation and visualization (Figure 5; Chignalia et al., 2016; Mitra et al., 2017; Harding et al., 2019). Due to its position, the GCX serves as a barrier to vascular permeability (Butler et al., 2020), can regulate the movement and absorption of blood-borne molecules, and can even affect the resistance to blood flow (Mitra et al., 2017; Zhu et al., 2018). These and other GCX functions are understandably largely dependent on the structural composition and stability of the GCX itself.

Glycoproteins of the GCX are protein-glycan conjugates that facilitate interactions with surrounding molecules and cells and include common adhesion molecules located at the base of the GCX. Three types of adhesion molecules play an important role in GCX structure: the selectin family, including E-selectin and P-selectin, which is highly associated with the interaction of leukocytes with the endothelium; the integrin family, which regulates platelet-EC interactions; and the immunoglobulin superfamily, which serve as ligands for integrins and additional mediators of adhesion to the endothelium (Figure 5; Mitra et al., 2017). Another major GCX component, proteoglycans, are core proteins to which many glycosaminoglycan (GAG) chains are covalently attached (Figure 5; Uchimido et al., 2019). Therefore, these proteins control the incorporation of extracellular GCX components into the EC body. The syndecan family of proteoglycans, which is composed of four members, is a unique transmembrane proteoglycan family involved in many biological processes like wound healing and inflammation (Chignalia et al., 2016). The glypican family, another proteoglycan family, is composed of six members and can be bound to glycosyl-phosphatidylinositol anchors within the cell membrane, which may contribute to its functional roles (Figure 5). The GAGs attached to these proteoglycans are composed of repeating disaccharide units and include heparan sulfate, which is the most common GAG on the GCX, chondroitin sulfate, which is believed to be the second most common GAG, and hyaluronic acid, which contributes to the structural integrity of the GAG-protein matrix. Additionally, GAGs and other GCX constituents are extensively modified with sialic acid residues, imparting a net negative charge to the structure. Collectively, these GCX components are highly associated with EC protection in the peripheral vasculature and several studies have even implicated the GCX as a critical component of the BBB, as discussed below (Ando et al., 2018).

### Endothelial Glycocalyx and BBB Function

Previous investigations in the peripheral vasculature, mainly using transmission electron microscopy, identified robust expression of the GCX *ex vivo* (Luft, 1966; van den Berg et al., 2003; Ueda et al., 2004; Ebong et al., 2011). Subsequent studies have implicated GCX of the peripheral vasculature in regulating key EC functions. For instance, abundant GCX expression has been shown to reduce endothelial permeability in multiple vascular beds, including the mesenteric capillaries and glomerular vasculature (Adamson, 1990; Singh et al., 2007). GCX expression in the peripheral vasculature has also been shown to mediate shear stress-induced increases in nitric oxide formation, which promotes vasodilation and in general is associated with vascular health (Ebong et al., 2014; Harding et al., 2018). Specifically, shear stress exposure was shown to increase endothelial nitric oxide synthase activation and expression in a heparan sulfate-dependent nature (Harding et al., 2018). However, whether the GCX is expressed at the BBB and, if so, its thickness and composition had until recently not been studied. The first confirmation of GCX expression in the BBB came in 2017 by Yoon et al. (2017) who identified GCX expression *in vivo via* fluorescence microscopy. By labeling the GCX with wheat germ agglutinin and performing 2-photon microscopy, which allows for *in vivo* imaging at sufficient depth, Yoon identified a fluorescent dextran permeation-resistant GCX layer of approximately 1 mm in thickness. In agreement with these findings, another investigation found that GCX within the BBB resists permeation of larger molecules, such as dextran, but not smaller molecules, such as fluorescein and Alexa Fluor antibodies (Kutuzov et al., 2018). The Yoon study also suggested that GCX expression varies in the cerebrovasculature, with the highest expression in arteries and no detectable expression in veins and venules. In 2018, Ando et al. (2018) validated the presence of the GCX in the BBB using electron microscopy. Specifically, transmission electron microscopy demonstrated a robust expression of the GCX in brain capillaries that was greater than the expression in the peripheral vasculature of similar diameter. Furthermore, the study determined that GCX of the BBB is more resistant to inflammation-induced degradation, which is known to occur in numerous indications. These results suggest that GCX integrity may play an even more significant role in regulating BBB function when compared to the peripheral vasculature.

In addition to these studies confirming the presence of the GCX *in vivo*, other studies have implicated the GCX in regulation of BBB function in physiological conditions. For example, DeOre et al. (2020) recently found that CD44, a hyaluronic acid binding protein, promotes BBB function by regulating BBB permeability in response to fluid shear stress. Specifically, CD44 knockdown led to increased permeability and decreased TEER after 24 h of flow exposure at 0.7 dynes/cm^2^, demonstrating the importance of CD44 expression in physiological conditions for proper mechanotransduction. In contrast to the beneficial effects of GCX expression in physiological conditions, GCX degradation has been identified in numerous pathologies, such as stroke and traumatic brain injury (TBI), suggesting that GCX degradation may play a functional role in the development of these conditions. For example, one study identified a degraded GCX layer in individuals with cerebral small vessel disease coinciding with white matter lesions (Martens et al., 2013). Another study in rats found that TBI significantly elevates levels of syndecan-1, a major GCX component, in serum samples compared to healthy controls (Jepsen et al., 2014). This is in agreement with other studies, including one using human subjects that identified increased syndecan shedding in response to TBI (Gonzalez Rodriguez et al., 2018). It was even found that repeated, low intensity TBI is sufficient to reduce both GCX thickness and density, which correlated with downstream behavioral deficits (Hall et al., 2017). GCX degradation is also associated with ischemia/reperfusion as occurs in ischemic stroke (Chappell et al., 2009; van Golen et al., 2012). Furthermore, a recent study by Zhu et al. (2018) found that hyaluronic acid degradation in rats *via* hyaluronidase treatment increases BBB permeability. While these studies demonstrate a correlation between GCX degradation and several neurological conditions, few studies have directly confirmed the role of the GCX in BBB function in physiological conditions. Furthermore, although GCX degradation is associated with ischemia/reperfusion as occurs in ischemic stroke, to our knowledge, no studies have extensively investigated the relationship between GCX expression and ischemic stroke pathology. Thus, future research should focus in these areas.

### Other Mechanotransducers Implicated in BBB Maintenance

In addition to the GCX, other endothelial mechanosensors are involved in the maintenance of BBB integrity and permeability. For example, it has been demonstrated that ion channels, particularly the transient receptor potential (TRP) channels, which are mechanosensitive, non-selective, calcium-permeable, cation channels, are heavily expressed on brain microvascular ECs (Chang et al., 2018) and serve as critical regulators of the intact BBB. Specifically, studies have demonstrated that transient receptor potential polycystin-2 (TRPP2; encoded by the PKD2 gene; AbouAlaiwi et al., 2009) and transient receptor potential channel 1 (TRPC1), which are responsible for calcium influx mediation, are highly involved in the response for BBB damage induced by TBI (Berrout et al., 2012). Both channels were suggested to modulate stretch-induced injury *via* nitric oxide (NO) production and action stress fiber formation in brain microvessel ECs. Marked decrease of the injury-induced calcium response, NO production, and stress fiber formation were observed after interfering with the function of TRPP2 and TRPC1 channel *via* TRPP2 and TRPC1 channel blockers or siRNA knockdown (Berrout et al., 2012). Ellaine et al. also demonstrated that stretch injury and oxygen glucose deprivation induced a significant increase in calcium ion concentration inside ECs (Salvador et al., 2015). Collectively, these studies suggest that ion channels may serve as potential therapeutic targets for BBB disruption. VE-cadherin has also been shown to regulate BBB function *via* endothelial mechanotransduction (Walsh et al., 2011). Specifically, Walsh et al. (2011) demonstrated for the first time that VE-cadherin transmits shear signals to occludin *via* Rac1, leading to BBB stabilization. This mechanosensory function of VE-cadherin likely occurs as part of the junctional mechanosensory complex, which has previously been shown to mediate EC responses to fluid shear stress (Conway et al., 2013).

### Endothelial Glycocalyx Regeneration as a Therapeutic Option

To date, limited therapies targeting GCX restoration or protection exist. However, recent *in vitro* and *in vivo* animal studies have demonstrated potential promise for GCX-targeted therapeutics aiming to restore endothelial and vascular function associated with endothelial mechanotransduction. For example, spingosine-1-phosphate (S1P), which is a membrane phospholipid metabolite, can protect against shedding of the GCX and induce biosynthesis of GCX components after their shedding (Zeng et al., 2015; Araibi et al., 2020). Specifically, Zeng et al. (2015) found that S1P induces the synthesis of syndecan-1 and incorporates heparan sulfate and chondroitin sulfate chains on ECs. This function was modulated by the phosphoinositide 3-kinase (PI3K) pathway. However, other findings have suggested that S1P induction of cardioprotection against ischemia-reperfusion injury is not relevant to the integrity of syndecan-1 in adult rats but instead *via* alternative S1P functions (Araibi et al., 2020). Future examination of the effects of S1P will provide greater insight into the mechanisms and potential usage of S1P for GCX recovery and potentially for conditions such as ischemic stroke associated with GCX degradation.

Another treatment strategy referred to as GAG replacement includes GCX repair through the administration of exogenous GAGs, such as heparan sulfate. These include sulodexide, which contains heparin (80%) and dermatan sulfate (20%), and can lead to the reconstruction of endothelium barrier function and inhibition of GCX degrading enzymes (Mitra et al., 2017). Similarly, experiments performed by our research group have shown that heparan sulfate-degraded rat ECs can be treated with exogenous heparan sulfate and S1P to restore GCX structure. Furthermore, not only was this treatment able to restore GCX structure, but it was also able to restore EC function, particularly cell-cell gap junction communication (Mensah et al., 2017). Taken together, these studies demonstrate the potential for GCX protection as a novel approach for treatment of diseases associated with vascular dysfunction. Future work further identifying the mechanisms of GCX-induced regulation of vascular function will corroborate the GCX as a target for vascular related disease. Combining these findings with the development of accurate therapeutics to protect or restore GCX expression may thus aid in the identification of GCX-based therapeutics for BBB protection or regeneration in diseases such as ischemic stroke.

## Conclusion

Over the past decade, our understanding of normal BBB and NVU functions and the mechanisms underlying BBB disruption during stroke has rapidly increased. It is well known that TJ proteins are complex and dynamic in nature and can be modulated in response to ischemic stroke while alterations of TJs can promote BBB permeability and oxidative stress-associated injury. In addition, endogenous BBB transporters further play a role in regulating BBB permeability and may be disrupted during ischemic stroke. Many therapeutic strategies have been investigated for their usage for ischemic stroke but with the exception of thrombolysis *via* recombinant tissue plasminogen activator (r-tPA) and endovascular mechanical thrombectomy treatment, these treatments have failed to pass clinical trials. New treatments targeting neuroprotection and neurorestoration, such as NBP and neurotrophins, respectively, are thus needed to help protect tissue injury from increasing in the setting of ischemic stroke. Additionally, endothelial mechanobiology, notably the endothelial GCX mechanotransducer, has been found to modulate BBB integrity and permeability, suggesting its high potential as a therapeutic target for BBB protection and recovery following ischemic stroke. However, the complete role of the GCX in BBB regulation has not been comprehensively studied, and few drugs have been developed for the protection or restoration of GCX components. Future research should further clarify the role of the GCX and other mechanotransducers in regulating BBB function and advance the development of novel therapeutics that target mechanotransducers in hopes of alleviating the impact of ischemic stroke.

## Author Contributions

KN, ICH, IMH, and EE were involved in the genesis of the review topic. KN and ICH drafted the manuscript. KN, ICH, EE, and IMH reviewed, edited, and approved the manuscript. All authors contributed to the article and approved the submitted version.

### Conflict of Interest

The authors declare that the research was conducted in the absence of any commercial or financial relationships that could be construed as a potential conflict of interest.

## Abbreviations

TJ: Tight junction
EC: Endothelial cell
AJ: Adherens junction
GCX: Glycocalyx
NBP: Dl-3-n-butylphthalide
Cop-1: Copolymer 1
GAG: Glycosaminoglycan.
